# School Start Times in Alabama

**DOI:** 10.1111/josh.70142

**Published:** 2026-04-01

**Authors:** Rebecca Williams, Anna M. Pyle, Caroline G. Richter, Krista Casazza, S. Justin Thomas, Heather Austin

**Affiliations:** ^1^ Department of Psychology University of Alabama at Birmingham Birmingham Alabama USA; ^2^ Virginia Commonwealth University Massey Cancer Center Richmond Virginia USA; ^3^ Department of Psychiatry & Behavioral Neurobiology University of Alabama at Birmingham Birmingham Alabama USA; ^4^ Division of Adolescent Medicine University of Alabama at Birmingham Birmingham Alabama USA

## Abstract

This commentary summarizes the evidence base, equity implications, and implementation considerations for later school start times (SSTs) in Alabama. In Alabama, almost half of children report sleep durations below age‐based recommendations. Of the 138 school districts in Alabama, few have middle and high schools adhering to the 8:30 am or later SSTs recommended by the American Academy of Sleep Medicine and the National Sleep Foundation. Historically designed to meet Industrial Revolution‐era schooling needs, current SSTs are largely dictated by tradition and fail to promote the health and well‐being of modern‐day students. Adolescent chronotype shifts later, producing later sleep onset and wake time. Given their naturally delayed chronotype, early SSTs often lead to poor sleep quality and shorter total sleep duration in adolescents, which can negatively impact metabolic and cardiovascular health. Earlier SSTs negatively impact social development as well. Earlier SSTs can result in shorter sleep duration, which has been associated with adolescent suicidal behavior and substance use. Early SSTs are also correlated with poorer academic achievement. The literature provides substantial evidence supporting a relation between later SSTs and improved adolescent physical and mental health, social development, academic outcomes, and decreased motor vehicle accident incidence. Given prolific racial/ethnic and socioeconomic disparities in Alabama, later SSTs may offer a practical and sustainable pathway for addressing health inequities in Alabama. The prioritization of the health, well‐being, and development of students warrants legislative discussion in Alabama regarding later start times for middle and high schools, along with strategic planning to overcome logistical challenges.

## 1 Introduction

In recent years, school start times (SSTs) have gained attention from developmental psychologists, parents, and legislators. In 2019, California passed Senate Bill 328, which dictated that middle schools should start no earlier than 8 a.m., and high schools no earlier than 8:30 a.m. [1]. Other states, including Connecticut, Indiana, Maryland, New Mexico, New Jersey, Pennsylvania, Utah, and Florida, have passed similar legislation, with eight other states introducing comparable bills [2]. At the federal level, the ZZZ's to A's Act (HR8787) was introduced in August, 2022 to conduct a study examining SSTs and adolescent health, well‐being, and performance [2]. This commentary summarizes the evidence base, equity implications, and implementation considerations for later SSTs in Alabama. In Alabama, 46.4% of children report sleep below age‐based recommendations [3]. The National Sleep Foundation (NSF) recommends 9–11 h of sleep for children age 6–13 years, and 8–10 h of sleep for children age 14–17 years [4]. In Birmingham, adolescents report shorter sleep on school nights and longer sleep on weekends, consistent with weekday sleep restriction [5, 6]. Of the 138 school districts [7] in Alabama, few have middle and high schools adhering to the 8:30 a.m. or later SSTs recommended by the American Academy of Sleep Medicine (AASM) and the NSF [8]. In Birmingham and Huntsville, high schools begin at 8:30 a.m., but middle schools begin at 8:00 a.m. [6, 9]. In Vestavia Hills, a wealthy suburb of Birmingham, no schools adhere to the AASM recommendation [10]. In Montgomery, only four of 52 public schools start at 8:30 a.m. or later [11]. Historically designed to meet Industrial Revolution‐era schooling needs, current SSTs are largely dictated by tradition and fail to promote the health and well‐being of modern‐day students. Thus, the issue of SSTs in Alabama is pressing.

Alabama students have shown high rates of chronic absenteeism since the COVID‐19 pandemic, with a rate of 18% in 2023 [12]. Though historically lower, the 2024 on‐time graduation rate for public high school students in Alabama reached 91% [13]. However, less than 50% of students who completed state assessments of math and science proficiency scored within higher proficiency levels in 2024 [14, 15]. Thus, Alabama students will still benefit from improved educational outcomes.

Importantly, sleep health may serve as a key marker of health equity and an avenue for intervention [16]. Alabama ranks high in poverty, with approximately 16% of Alabamians earning less than the poverty threshold [17]. Rates are highest among Black/African American and Hispanic or Latine residents, with 25% experiencing poverty [18]. Related to poverty, in 2023, Alabama had the fourth highest rates of child obesity in the country [19]. Notably, evidence suggests that socioeconomically disadvantaged students in particular may benefit academically from delayed SSTs [18]. Given prolific racial/ethnic and socioeconomic disparities in Alabama, later SSTs may offer a practical and sustainable pathway for addressing health inequities in Alabama.

## 2 Adolescent Chronotype

Adolescent chronotype shifts later, producing later sleep onset and wake time. This normative shift is driven by circadian timing and homeostatic sleep processes rather than pathology [20, 21, 22]. The adolescent chronotype results from biologically occurring changes in homeostatic and circadian processes, along with broader neurodevelopmental and behavioral changes as well as sociocultural and environmental cues [23]. For example, evidence suggests that residing in urban areas, living with a smoker, and higher parental education are linked to higher eveningness among adolescents [24]. Further, the siesta tradition among Spanish adolescents is an example of a cultural factor affecting tendencies toward eveningness [25]. Carskadon and colleagues [26] also analyzed self‐report data from 448 sixth graders and observed that children further along in puberty had later bedtimes. Pubertal changes are also linked with maturational changes in sleep, including later bedtimes [27, 28], suggesting a biological underpinning.

## 3 Impact of Early School Start Times

Given their naturally delayed chronotype, early SSTs often lead to poor sleep quality and shorter total sleep duration in adolescents, which can negatively impact metabolic and cardiovascular health. One study with 1390 students in Philadelphia found that each additional hour of sleep was associated with a 0.28, 0.17, and 0.07 kg/m^2^ reduction in BMI at the 90th, 50th, and 10th BMI percentiles, respectively [29]. Additionally, sleeping ≤ 7 h has been associated with higher blood pressure, and sleeping < 5 h is similarly linked with a greater risk for some cardiometabolic risk factors among adolescents [30, 31, 32]. Somatic outcomes are also positively associated with poor sleep in adolescents [33]. Altogether, early SSTs can have a deleterious impact on adolescent health.

Earlier SSTs may negatively impact social development as well. Using data from 718 public elementary schools in Kentucky (*n* = 310,470), one study found that later SST was related to less harassment and fewer removals, suspensions, and expulsions [34]. Misaligned sleep schedules are associated with increased behavioral difficulties, poorer peer relationships, and greater victimization across time [24, 35, 36, 37]. Evidence also suggests that delayed SSTs can reduce behavioral and verbal aggression among adolescents [38].

Earlier SSTs can result in shorter sleep duration, which has been associated with adolescent suicidal behavior and substance use [39, 40]. Wolfson and Carskadon [41] also found that among 3120 high school students, those with later bedtimes and shorter sleep times had worse grades and more depressed moods.

The transition into daylight saving time each spring exacerbates the negative effect of early SSTs on adolescent affective development. Schneider and Randler [42] surveyed 469 German adolescents and found that eveningness chronotype increased with age and that daytime sleepiness was higher > 3 weeks after the daylight saving transition. Indeed, year‐round daylight saving time, adopted in 1973, was abolished just 2 years later after a rise in traffic fatalities among school‐age children during dark winter mornings [43, 44]. In Alabama, the sun rises shortly after 7:00 a.m. instead of 6:00 a.m. after the transition from standard time to daylight saving time, meaning most students expected at school by 8:00 a.m. are waking up in the dark. Waking up in the dark can exacerbate sleepiness, as sunlight signals the internal clocks to cease melatonin production. Thus, an elimination of daylight saving time would help reduce the negative impacts of early SSTs on adolescent development.

Early SSTs are also negatively correlated with academic achievement. In a quasi‐experiment conducted within high schools in North Carolina, Lenard and colleagues [35] found that after adopting earlier SSTs, they observed no effect on ACT scores but observed 11% higher absenteeism, 50% higher tardiness, and 1.2% higher drop‐out rates. Impaired cognition and poorer grades are also linked with earlier SSTs, suggesting that earlier SSTs negatively impact academic achievement [36].

## 4 Impact of Later School Start Times

Later SSTs allow adolescents to sleep at times that align with their biological inclination. Rogers and colleagues [37] found that adolescents had later bedtimes, later rise times, less sleepiness, and marginally longer total sleep time during the COVID‐19 pandemic than before the pandemic. Owens and colleagues [45] (*n* = 201) also found that delaying SST by 30 min increased high school students' total sleep time by 45 min and reduced fatigue and daytime sleepiness. After experiencing the benefits of a later wake time, it was posited that students may adapt their sleep–wake routines to further increase sleep duration, reinforcing the downstream advantages to later SSTs.

The authors also found decreased visits to the school health center for fatigue‐related complaints after the SST delay, as well as reduced depressed mood. Additionally, in Fairfax County, Virginia, researchers [40] found that 50‐ and 30‐min delays in SSTs across 16 public schools (*n* = 1180 after the change) decreased daytime sleepiness by 4.8% and increased total sleep time by approximately 35 min, all in all suggesting improved physical and affective functioning in adolescents.

Academically, one study [45] observed a 45% decrease in class absences and tardies after a 30‐min delay in SST. Another study [46] reported a 4% increase in class attendance and a 9% increase in graduation rates after nine high schools across seven states (*n* > 30,000) implemented an SST later than 8:30 a.m. Bastian and Fuller [47] analyzed data from disadvantaged youth in North Carolina and found that delaying SST by one hour was associated with a 1.3% decrease in students' likelihood of suspension.

Groen and Pabilonia [48] found that students slept more at night at schools with later SSTs and that female (but not male) students scored higher on reading tests. Wahlstrom et al. [49] analyzed data from > 9000 students in eight public high schools and reported improvements in academic performance and attendance among adolescents with later SSTs. One study observed changes after delaying SST by an hour in a Seattle School District and found that grades improved by 4.5% and tardies and absences went down, especially among socioeconomically disadvantaged students [18].

Student athletes may also benefit from later SSTs. Among collegiate men's basketball players, increasing sleep duration over a five‐ to seven‐week period led to a 9% improvement in free‐throw accuracy, a 0.7‐s reduction in sprint time, and overall improvements in both mental and physical well‐being [50]. Additional research [51] suggests that adolescent athletes who obtain at least eight hours of sleep are less likely to sustain injuries compared to sleep‐deprived peers, highlighting the benefits of delayed SSTs for supporting athletic performance and safety.

Perhaps most critical of the changes reported in adolescent health outcomes after SST changes is the decrease in motor vehicle accidents (MVA). According to the Centers for Disease Control and Prevention (CDC), approximately eight teenagers die from MVA each day in the United States [52]. Youth.gov reported that MVAs are the leading cause of death among United States teenagers [53]. One high school that changed its SST from 7:35 to 8:55 a.m. reported a 70% reduction in MVAs among its driving students, which has been supported by additional studies [54, 55].

## 5 Future Directions

Much of the literature relies on self‐report sleep measures and nonrandomized designs, increasing the risk of recall bias and confounding [56]. Many sleep disorders, however, are diagnosed and treated based on self‐report. Thus, comprehensive examinations of SST impacts should utilize subjective and objective sleep measures. Future research should consider examining the impacts of different SSTs on sleep quality and architecture as measured with objective sleep measures, like electroencephalography and polysomnography, which have demonstrated high feasibility and acceptability [57].

## 6 Conclusions and Recommendations

The literature provides substantial evidence supporting a relation between later SSTs and improved adolescent physical and mental health, social development, academic outcomes, and decreased MVAs. In 2017, the AASM called on school boards to implement SSTs no earlier than 8:30 a.m. for all middle and high schools [58]. The American Academy of Pediatrics [59], the CDC [60], and the American Medical Association [61] have also issued statements supporting SSTs of 8:30 a.m. or later among other groups [62]. Such recommendations are developed through transparent, evidence‐based processes [61, 63, 64]. At least 17 states have introduced or passed laws in response to this AASM recommendation [2]. The prioritization of the health, well‐being, and development of its students warrants Alabama legislation discussion for middle and high schools to start later, with strategic planning for overcoming logistical challenges.

Serving 36,000 students, Jefferson County School District is one of the largest school districts in the state of Alabama, boasting a fleet of over 500 buses transporting nearly 19,000 students per day. Delayed SSTs may lead to traffic congestion in the morning and afternoon, increasing the likelihood of late arrivals for both students and working parents [65]. The potential mismatch between SSTs and parent work schedules may also make mornings more logistically challenging. Table 1, adapted from Barnes et al. [66], addresses some of the potential barriers to SSTs changes. Implementation plans at the district level vary across communities, meaning barriers and solutions may exist at different capacities within individual school systems. Thus, consideration of local infrastructure, resources, and stakeholder perspectives is essential when adopting delayed SSTs.

**TABLE 1:** National Sleep Foundation's barriers and solutions to later school start times.

Barrier	Concerns	Solution 1	Solution 2
Transportation	Traffic congestion for both students and teachers at later times	Flip elementary and high school start times	Shift to public transportation for high school routes
Extracurriculars	Students who have after school jobs or those who have non‐school‐directed activities	Reschedule practice and games for later in the day	Install extra lighting for later extracurriculars
Impact on other students/programs	May require additional planning for special education students and career centers	Advance morning childcare schedules	Assign parents on rotating schedule as neighborhood bus stop supervisors
Reduced time to access public resources	No solutions offered though it is hypothesized that students with better sleep may be more efficient workers and thus this will not be as much of a concern		
Teachers	Teachers with students whose schedules may no longer match their own	Alter “planning time” to early morning so that teacher schedules do not change	Implement early morning childcare at schools as needed
Parent work schedules	Parents' work schedules may not be able to accommodate changes	Encourage carpooling networks to assist with transportation gaps	Work with employers to raise awareness about benefits of later SSTs and encourage flexible work hours for parents (e.g., remote or hybrid work options, delayed work start times)
Family stress	Most families schedules are finely tuned and thus a disruption can be overwhelming. Community involvement is key	Involve families in schedule planning ahead of implementation of new school start times	Implement hotlines, message boards, and meetings to increase communication and problem‐solving
Uneducated community	Lack of knowledge in the community may decrease community buy‐in	Campaign in local community to increase awareness of changes and reasons behind the change	Educate community about negative outcomes related to sleep deprivation, not just for students but for everyone
Student resistance	Students can be resistant to change even if beneficial	Educate students about benefits of new start time. Can be done in relevant classes	Include students in early discussions to gain support

*Note:* Adapted with permission from Barnes et al. [66, p. 4].

It is clear that difficult logistical changes are worth making to protect the health and well‐being of adolescents in Alabama. Addressing logistics will require significant school district decisions, schedule changes, and legislative effort, but for nearly two centuries, school schedules have been built around antiquated needs of the Industrial Revolution. It is time to pass the legislation needed for later SSTs in Alabama and promote the health and well‐being of modern‐day adolescents.

## Funding

This work was supported by the Foundation for Children with Intellectual and Developmental Disabilities, the Agency for Healthcare Research and Quality (T32HS013852).

## Ethics Statement

The authors have nothing to report.

## Conflicts of Interest

The authors declare no conflicts of interest.

## Data Availability

Data sharing not applicable to this article as no datasets were generated or analysed during the current study.
